# Mutation detection analysis of a region of 16S-like ribosomal RNA gene of *Entamoeba histolytica*, *Entamoeba dispar *and *Entamoeba moshkovskii*

**DOI:** 10.1186/1471-2334-8-131

**Published:** 2008-09-26

**Authors:** Subhash Chandra Parija, Krishna Khairnar

**Affiliations:** 1 Department of Microbiology, Jawaharlal Institute of Postgraduate, Medical Education and Research, Puducherry, 605006, India

## Abstract

**Background:**

The level of intra-species genetic variation in *Entamoeba histolytica, Entamoeba dispar *and *Entamoeba moshkovskii *populations in a localized geographic area, like Puducherry, India, remains unknown.

**Methods:**

In the present study the existence of genetic variation in the nested multiplex polymerase chain reaction (NM-PCR) amplified region of the 16S-like ribosomal RNA genes of *E. histolytica, E. dispar *and *E. moshkovskii *was investigated by riboprinting and single strand conformation polymorphism (SSCP) analysis.

**Results:**

We found that 70 stool specimens were positive for *E. histolytica*, 171 stool specimens were positive for *E. dispar*, and 37 stool specimens were positive for *E. moshkovskii *by NM-PCR. Ninety liver abscess pus specimens, 21 urine specimens, and 8 saliva specimens were positive for *E. histolytica *by NM-PCR. Riboprinting analysis detected a mutation in the PCR product of only one *E. histolytica *isolate from a stool specimen. However, SSCP analysis detected mutations in the PCR products of five *E. histolytica *isolates and three *E. moshkovskii *isolates from stool specimens, and one *E. histolytica *isolate from a saliva specimen. The mutations detected by riboprinting and SSCP analysis were confirmed by sequencing. All the nucleotide sequences showing mutations in this study have already been deposited into the NCBI GenBank database under accession numbers [GenBank: EF682200 to GenBank: EF682208].

**Conclusion:**

The present study has revealed the subsistence of mutations in the ribosomal RNA genes of *E. histolytica *and *E. moshkovskii*, which points towards the existence of intra-species genetic variation in *E. histolytica *and *E. moshkovskii *isolates infecting humans.

## Background

The protozoan parasite *Entamoeba histolytica *is estimated to infect 50 million people and cause 40,000 to 100,000 deaths annually, making it the second largest cause of mortality from infection with parasitic protozoa after malaria [1].

Although the first description of amoebiasis was more than a century ago [2], there is still uncertainty as to why symptoms of the disease appear only in 10% of those infected with *E. histolytica *while majority remains asymptomatic [3]. There have been several suggestions regarding the factors that may contribute to the outcome of amoebic infection in a susceptible host, which include a range of virulence levels among the *E. histolytica *strains and variability in host immunity against amoebic invasion. While the variability of human immunity against amoebic infection is not well understood, the existence of genetic variation in *E. histolytica *has been studied in depth recently [4-17]. These studies have identified genetic variation in protein-coding sequences of *E. histolytica*, such as those for the serine-rich *E. histolytica *protein [10-15] and chitinase [8,11,12], as well as non-protein-coding regions such as the ribosomal RNA (rRNA) genes [4,5,7] and loci 1–2 and 5–6 [11,12,16,17]. In addition, the existence of genetic variation in non-protein-coding loci 1–2 and 5–6 [18], as well as protein-coding chitinase gene of *E. dispar *has been reported recently [19].

These genetic variation studies appear to be promising in investigating the molecular epidemiology of amoebiasis. The existence of significant genetic variation among *E. histolytica *isolates collected from a wide geographical range, including Mexico, Bangladesh, India, Venezuela, South Africa, the Philippines, and Georgia, has already been demonstrated [8,9,13,14]. However, whether intra-species genetic variation also exists in *E. histolytica, Entamoeba dispar *and *Entamoeba moshkovskii *from a population in a restricted geographic area like Puducherry, India, still remains unknown.

The rRNAs, especially the 16S rRNA, have been widely used for studying genetic variation because of their conservative nature and universal distribution [20]. In the present study an attempt has been made to study genetic variation in regions of the 16S-like rRNA gene of *E. histolytica*, *E. dispar *and *E. moshkovskii *using riboprinting and single strand conformation polymorphism (SSCP) analysis followed by confirmation by nucleotide sequencing.

## Methods

### Sample details

The study was conducted at the Jawaharlal Institute of Postgraduate Medical Education and Research (JIPMER) hospital, Puducherry, India, during the period from July 2004 to July 2006. Informed consent was obtained from the patients. The study was approved by the Institute Human Ethics Committee (JIPMER, Puducherry, India).

#### Stool

Fresh unpreserved stool samples from 202 patients with complaints of gastrointestinal discomfort and positive for *E. histolytica*, *E. dispar*, or *E. moshkovskii *by microscopy or culture were collected in sterile capped containers and stored at -20°C until used.

#### Liver abscess pus

The liver abscess pus aspiration was performed only for clinical purposes, as judged necessary by the clinicians for the patient care and not for the purpose of this study. The liver abscess pus was obtained under ultrasound guidance from 112 amoebic liver abscess (ALA) patients and stored at -20°C in a sterile container until used.

#### Urine

A urine specimen was collected from 53 ALA patients. Ten millilitres of urine were collected in a sterile container using aseptic techniques and stored at -20°C until used.

#### Saliva

A saliva specimen was collected from 28 ALA patients. Five millilitres of saliva were collected in a sterile container using aseptic techniques and stored at 4°C until used.

### *Entamoeba *16S-like rRNA gene amplification by nested multiplex polymerase chain reaction (NM-PCR)

#### Extraction of *Entamoeba *genomic DNA

The extraction of *Entamoeba *genomic DNA from stool, liver abscess pus, urine, and saliva specimens was performed as per the method described previously [21-23].

#### Primers used

Based on the sequences of the 16S-like rRNA gene of *E. histolytica*, *E. dispar *and *E. moshkovskii*, nested sets of primers (designated E-1/E-2, EH-1/EH-2, ED-1/ED-2 and Mos-1/Mos-2) were used as described previously [21].

#### Standard strains

*E. histolytica *HM-1:IMSS, *E. dispar *SAW760, and *E. moshkovskii *Laredo were the standard strains used as positive controls in the present study. The lyophilized DNA of these strains was generously gifted by Dr. C. Graham Clark from the London School of Hygiene and Tropical Medicine, London, UK.

### NM-PCR

#### Stool PCR

The PCR mix composition was as described previously [21].

#### Liver abscess pus PCR

For genus specific and species specific PCR, the reaction volume of 25 μl was comprised of 2.5 μl of 10× PCR buffer (Biogene), 2.0 μl of 25 mM MgCl_2 _(Bangalore genei), 0.75 μl of deoxyribo-nucleotide triphosphate mix (10 mM each dNTP, Biogene), 0.3 μl (5 IU/μl) of *Taq *polymerase (Biogene), 0.25 μM primers (IDT) and 2.0 μl of template DNA. Amplification was performed in an Eppendorf Thermal cycler [Master cycler gradient].

#### Urine PCR

The PCR mix composition was the same as described above for liver abscess pus PCR, except that 1.0 μl of 25 mM MgCl_2 _and 2.5 μl of template DNA was added.

#### Saliva PCR

The PCR mix composition was the same as described above for liver abscess pus PCR, except that 1.0 μl of 25 mM MgCl_2 _was added.

The PCR cycle conditions were as described previously [21].

### Mutation detection by riboprinting

#### Restriction enzymes

Restriction enzymes were used to generate comparative riboprints. The restriction enzymes were selected for the 439 bp, 553 bp and 174 bp PCR product of 16S-like rRNA gene of *E. histolytica*, *E. moshkovskii *and *E. dispar*, respectively, using the online software "NEB cutter V2.0" . The restriction enzymes used are shown in Table 1.

**Table 1:** Restriction enzymes used for riboprinting analysis of partial regions of the 16S-like rRNA genes of *E. histolytica*, *E. dispar *and *E. moshkovskii*

*E. histolytica *(439 bp)	*E. moshkovskii *(553 bp)	*E. dispar *(174 bp)
*Xba *I	*Taq *I	*Taq *I
*Dde *I	*Dde *I	*Dde *I
*Hpy*188 III	*Hpy*188 III	*Mse *I
*Mfe *I	*Mfe *I	*Sau*96 I
*N1a*IV	*N1a*IV	
*Hin*f I	*Hin*f I	
*Mse *I	*Mse *I	
	*Sau*96 I	

#### Restriction enzyme digestion

In a 25 μl reaction mix, 15 μl of nested PCR product was digested with 2.5 units of each restriction enzyme in separate 0.2 ml tubes. The incubation period was for 16 hrs at 37°C except for the restriction enzymes *Taq *I and *Mse *I where the incubation temperature was 65°C.

#### Agarose gel electrophoresis

Twenty microlitres of the restriction enzyme digested PCR product was separated by electrophoresis through a 2.5% agarose gel (Agarose Low EEO, Bangalore genie products, Bangalore, India) containing ethidium bromide in 0.5 × Tris-acetate-EDTA (TAE) buffer at 120 V for 45 min and was visualized under UV light.

### Mutation detection by SSCP

#### Preparation of the SSCP gel

In the present study 6% and 9% SSCP gels were used. Six percent SSCP gels were used for mutation detection in the 439 bp and 553 bp PCR products of *E. histolytica *and *E. moshkovskii*, respectively. A nine percent SSCP gel was used for mutation detection in the 174 bp PCR product of *E. dispar*.

#### Preparation of samples for SSCP electrophoresis

Two microlitres of the nested PCR product was diluted into 20 μl formamide dye mix in a 0.2 ml microfuge tube. A similar aliquot was diluted into 20 μl of gel loading dye. The formamide containing sample was boiled for 6 minutes in a water bath, and then the tube was plunged directly into ice for 10 minutes.

#### Separation of DNA fragments by SSCP gel electrophoresis

The wells of the polyacrylamide gel were washed with 1× TAE electrophoresis buffer. 4 μl of denatured DNA (DNA boiled with formamide dye mix) was loaded into the well of polyacrylamide gel. Two microlitres of non-denatured DNA (DNA with gel loading dye) was loaded into another well. Approximately 6–7 V/cm (~110 V [and 16 mA] for 18 × 14-cm gel) was applied to the gel for 16 hrs at a temperature of 20–25°C in an air conditioned room. On completion of the electrophoresis, the gel was silver stained.

#### Silver staining of SSCP gel

Silver staining for nucleic acid was performed as per the method described previously [20]. The gel was documented using a densitometer (Biorad) and the results analyzed using "Quantity One" software (Biorad). For long term storage the gel was kept in 10% glycerol.

### Nucleotide sequencing

PCR products of 16S-like rRNA genes of *E. histolytica*, *E. dispar *and *E. moshkovskii *showing mutation by riboprinting or SSCP analysis, were sequenced on a ABI3730XL sequencer (Macrogen, Seoul, South Korea) using species specific primers. The nucleotide sequences were edited with reference to chromatographs using Chromas (Version 1.62) software and aligned using BioEdit (version 7).

### Nucleotide sequence accession numbers

The nucleotide sequences showing mutations in this study have been deposited into the NCBI GenBank database under accession numbers EF682200–EF682208] (Table 2).

**Table 2:** *E. histolytica *and *E. moshkovskii *isolates obtained from clinical specimens of patients attending JIPMER hospital, Puducherry, showing mutation by PCR-SSCP analysis

S. No	*Entamoeba *species	Clinical specimen	GenBank submission [GenBank accession number]
1	*E. histolytica*	Stool	*Entamoeba histolytica *isolate 1 [GenBank: EF682200]
2	*E. histolytica*	Stool	*Entamoeba histolytica *isolate 2 [GenBank: EF682201]
3	*E. histolytica*	Stool	*Entamoeba histolytica *isolate 3 [GenBank: EF682202]
4	*E. histolytica*	Stool	*Entamoeba histolytica *isolate 4 [GenBank: EF682203]
5	*E. histolytica*	Stool	*Entamoeba histolytica *isolate 5 [GenBank: EF682204]
6	*E. moshkovskii*	Stool	*Entamoeba moshkovskii *isolate 6 [GenBank: EF682206]
7	*E. moshkovskii*	Stool	*Entamoeba moshkovskii *isolate 7 [GenBank: EF682207]
8	*E. moshkovskii*	Stool	*Entamoeba moshkovskii *isolate 8 [GenBank: EF682208]
9	*E. histolytica*	Saliva	*Entamoeba histolytica *isolate 1 [GenBank: EF682205]

## Results

### *Entamoeba *16S-like r RNA gene amplification by NM-PCR

#### Stool PCR

Seventy stool specimens were positive for the 439 bp PCR product of *E. histolytica*, 171 stool specimens were positive for the 174 bp PCR product of *E. dispar*, and 37 stool specimens were positive for the 553 bp PCR product of *E. moshkovskii*.

#### Liver abscess pus PCR

Ninety liver abscess pus specimens were positive for the 439 bp PCR product of *E. histolytica*.

#### Urine PCR

Twenty one urine specimens were positive for the 439 bp PCR product of *E. histolytica*.

#### Saliva PCR

Eight saliva specimens were positive for the 439 bp PCR product of *E. histolytica*.

### Mutation detection by riboprinting

No intra-species variation in restriction fragment length polymorphism (RFLP) patterns for any restriction enzyme was observed, except for the *N1a*IV RFLP pattern of the *E. histolytica *specific 439 bp PCR product from one stool specimen (Stool- 5).

**Figure 1:**
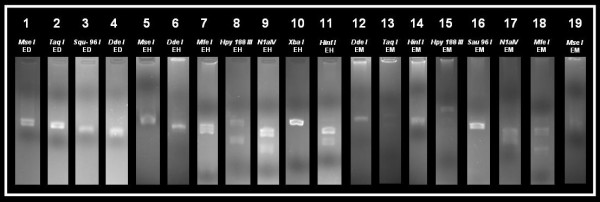
**Normal RFLP digestion pattern for 174 bp, 439 bp, and 553 bp PCR products of *E. dispar*, *E. histolytica *and *E. moshkovskii *respectively with various restriction enzymes.** Lane-1 to 4 normal riboprints for *E. dispar *(ED); Lane-5 to 11 normal riboprints for *E. histolytica *(EH); Lane-12 to 19 normal riboprints for *E. moshkovskii *(EM).

Normal RFLP digestion pattern for the 174 bp, 439 bp, and 553 bp PCR product of *E. dispar*, *E. histolytica *and *E. moshkovskii *respectively with various restriction enzymes are shown in figure 1.

### Mutation detection by SSCP

**Figure 2:**
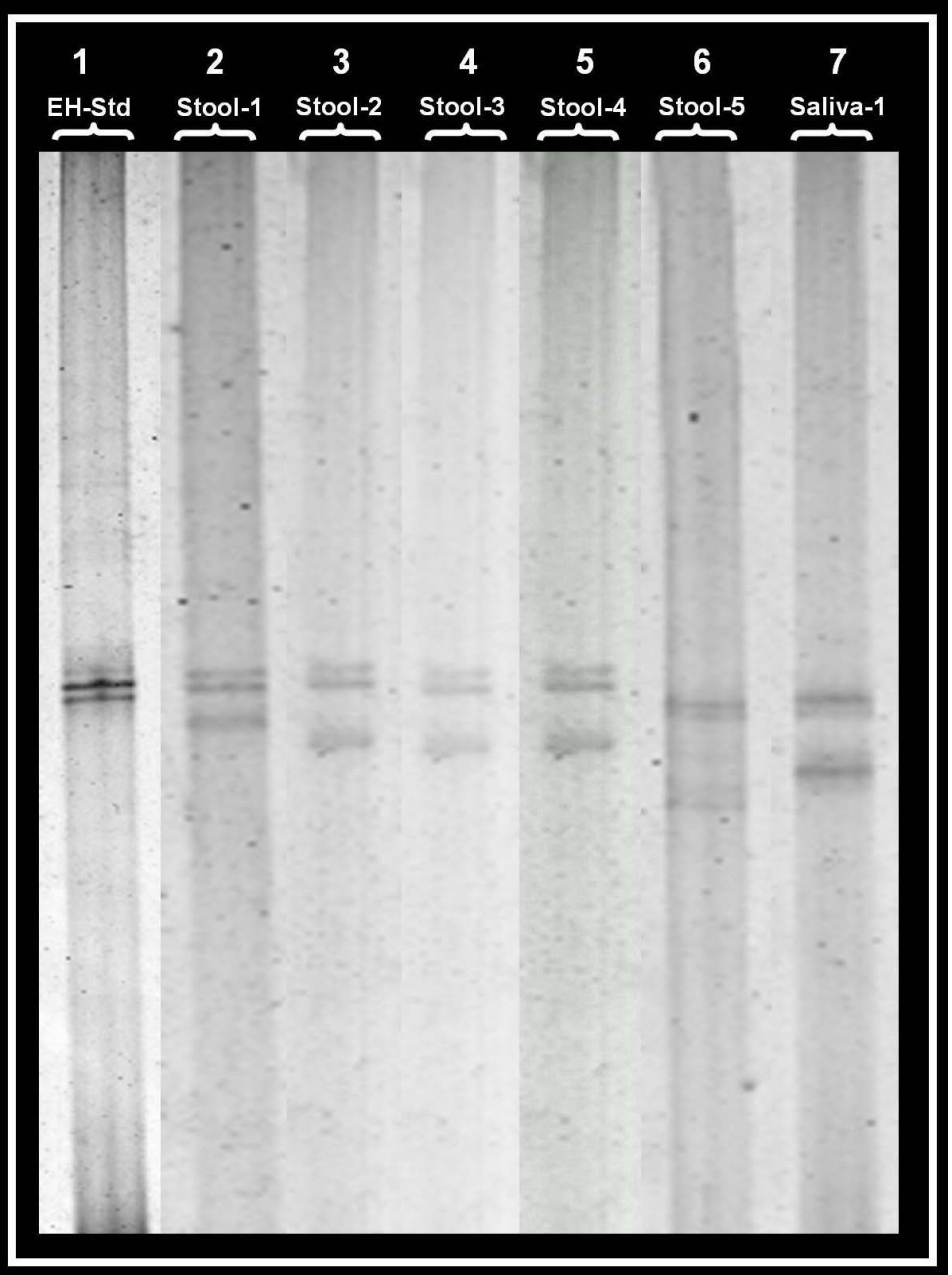
**SSCP analysis showing altered profile (mobility shift) for the 439 bp PCR product of *E. histolytica*.** Lane-1 is for *E. histolytica *standard DNA (EH-Std); Lane-2, 3, 4, 5, and 6 are for *E. histolytica *DNA from stool specimens showing an altered profile; Lane-7 is for *E. histolytica *DNA from a saliva specimen showing an altered profile.

**Figure 3:**
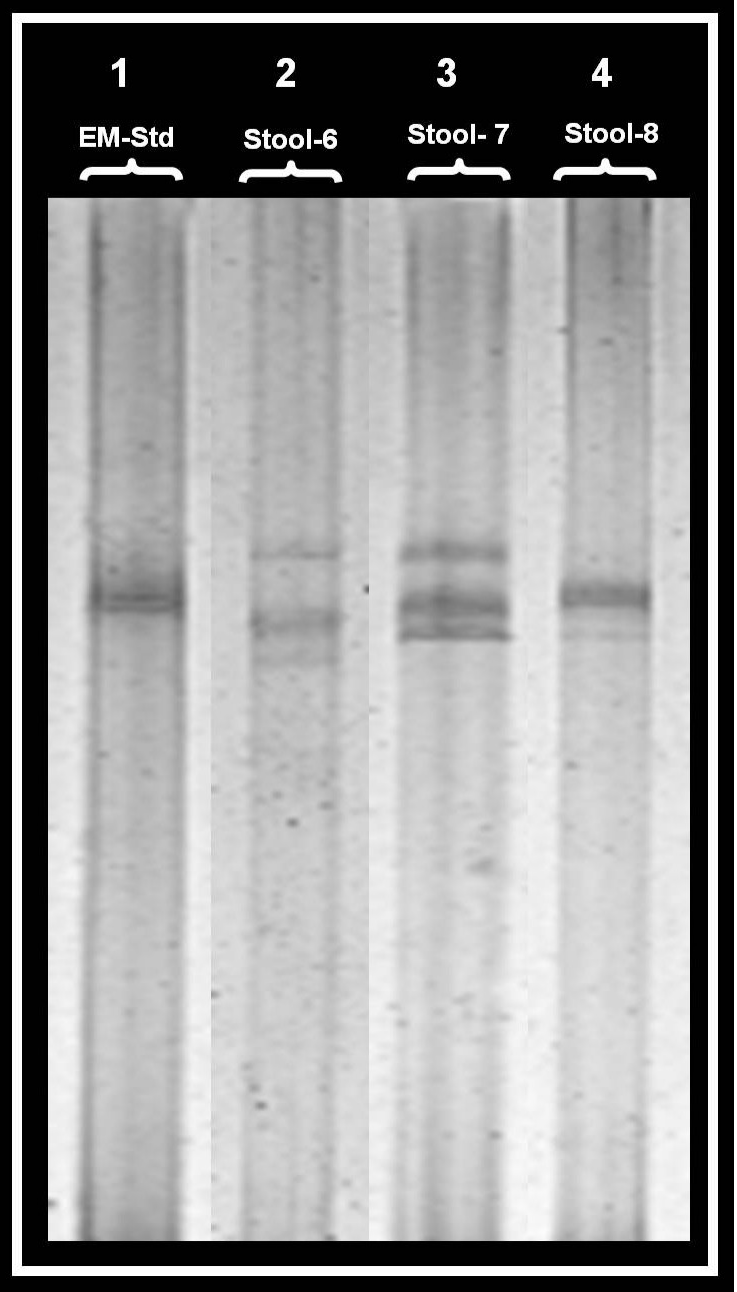
**SSCP analysis showing altered profile (mobility shift) for the 553 bp PCR product of *E. moshkovskii*. Lane-1 is for *E. moshkovskii *standard DNA (EM-Std);** Lane-2, 3, and 4 are for *E. moshkovskii *DNA from stool specimens showing an altered profile.

Five out of 70 *E. histolytica *specific 439 bp PCR products from stool specimens, one out of 8 *E. histolytica *specific 439 bp PCR products from saliva specimens, and three out of 37 *E. moshkovskii *specific 553 bp PCR products from stool specimens showed an altered SSCP profile (i.e. a mobility shift in the acrylamide gel) suggestive of a mutation (Figure 2 and Figure 3).

None of the 171 *E. dispar *specific 174 bp PCR products from stool specimens, 90 *E. histolytica *specific 439 bp PCR products from liver abscess pus specimens, or 21 *E. histolytica *specific 439 bp PCR products from urine specimens showed an altered SSCP profile suggestive of a mutation.

The *E. histolytica *and *E. moshkovskii *isolates showing mutations by PCR-SSCP analysis are summarized in table 2.

### Nucleotide sequencing

**Figure 4:**
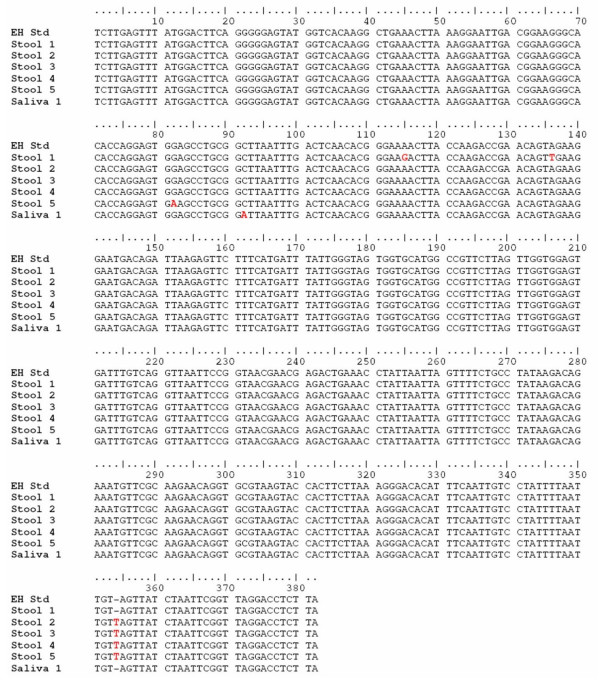
**Multiple sequence alignment of *E. histolytica *specific 439 bp PCR products from clinical specimens (Stool-1 to 5 and Saliva-1) and the standard strain *E. histolytica *HM-1:IMSS (EH-Std).** The sequence variations are highlighted in red.

**Figure 5:**
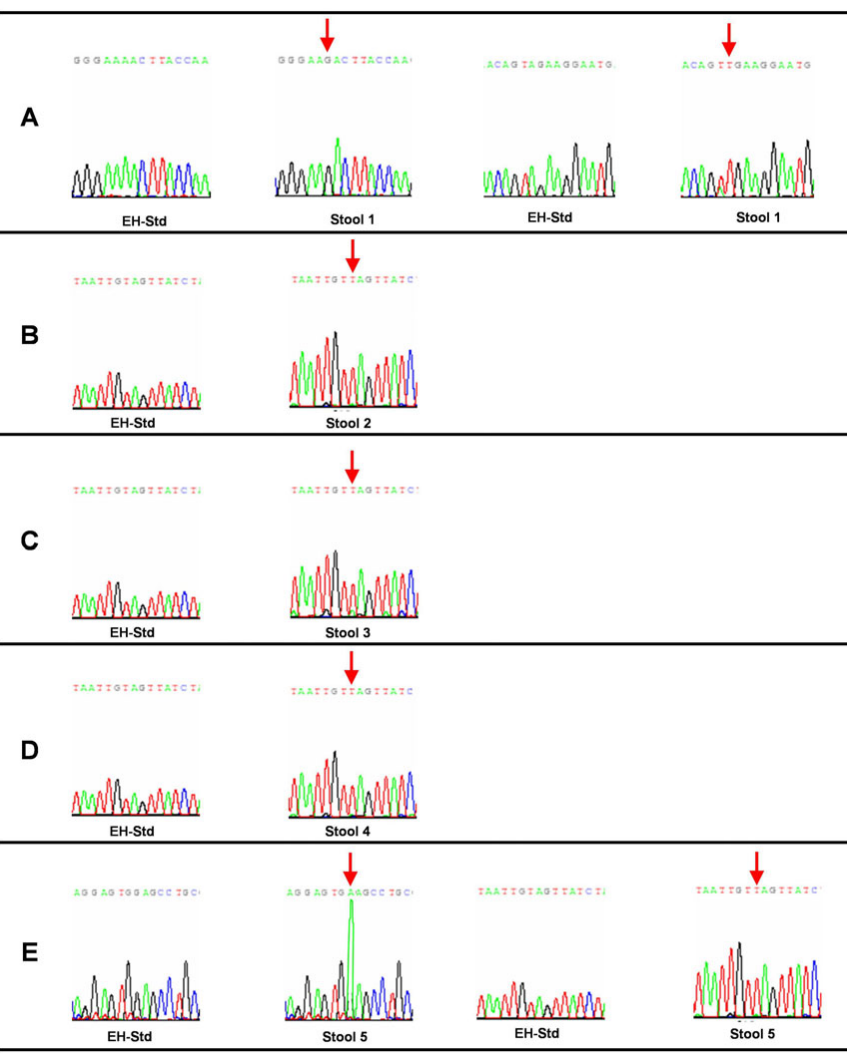
**Electropherogram showing the mutant (Stool-1 to 5) and normal control *E. histolytica *HM-1:IMSS (EH-Std) 16S-like rRNA genes. **Chromatogram showing the sequence variation is indicated by an arrow. The sequencing revealed substitution of A by G and substitution of A by T (A), insertion of T (B), insertion of T (C), insertion of T (D), and substitution of G by A and insertion of T (E).

All four *E. histolytica *specific 439 bp PCR products from stool specimens (Stool- 1, 2, 3, and 4) showing a mutation by SSCP analysis and the *E. histolytica *specific 439 bp PCR product from stool specimen showing mutation by both SSCP and riboprinting analysis (Stool- 5), were confirmed by nucleotide sequencing. The multiple sequence alignment of these sequencing results and the standard strain *E. histolytica *HM-1:IMSS is depicted in figure 4. The comparative electropherogram showing the mutant (Stool- 1, 2, 3, 4, and 5) and normal control (*E. histolytica *HM-1:IMSS) 16S-like rRNA genes is shown in figure 5.

**Figure 6:**
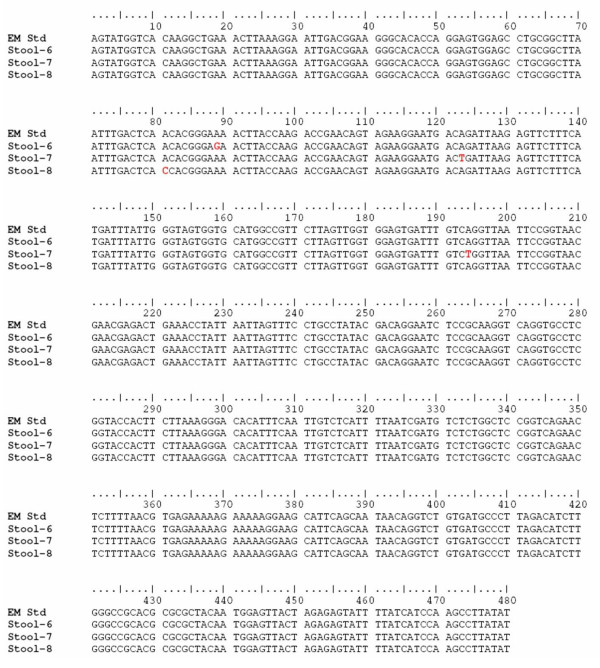
**Multiple sequence alignment of the *E. moshkovskii *specific 553 bp PCR products from stool specimens (Stool-6 to 8) and the standard strain *E. moshkovskii *Laredo (EM-Std)**. The sequence variations are highlighted in red.

**Figure 7:**
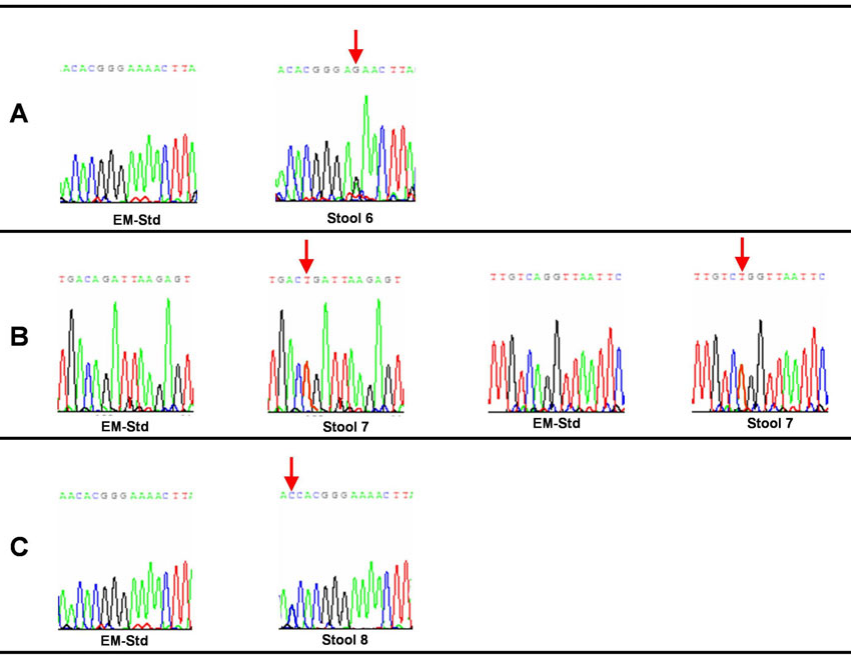
**Electropherogram showing the mutant (Stool-6 to 8) and normal control *E. moshkovskii *Laredo (EM-Std) 16S-like rRNA genes.** Chromatogram showing the sequence variation is indicated by an arrow. The sequencing revealed substitution of A by G (A), substitution of A by T and substitution of A by T (B), and substitution of A by C (C)

All three *E. moshkovskii *PCR products showing mutation by SSCP analysis (Stool- 6, 7, and 8) were confirmed by nucleotide sequencing. The multiple sequence alignment of these sequences and the standard strain *E. moshkovskii *Laredo is depicted in figure 6. The comparative electropherogram showing the mutant (Stool- 6, 7, and 8) and normal control (*E. moshkovskii *Laredo) 16S-like rRNA genes is shown in figure 7.

**Figure 8:**
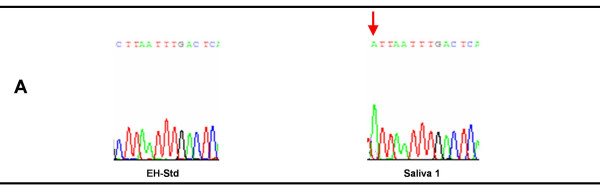
**Electropherogram showing the mutant (Saliva-1) and normal control *E. histolytica *HM-1:IMSS (EH-Std) 16S-like rRNA genes.** Chromatogram showing the sequence variation is indicated by an arrow. The sequencing revealed substitution of C by A (A)

The one *E. histolytica *PCR product from a saliva specimen showing mutation by SSCP analysis was also confirmed by sequencing. The multiple sequence alignment of the results from this saliva specimen and the standard strain *E. histolytica *HM-1:IMSS is depicted in figure 4. The comparative electropherogram showing the mutant (Saliva-1) and normal control (*E. histolytica *HM-1:IMSS) is shown in figure 8.

## Discussion

Mutations and other polymorphisms in genes, gene systems, or whole genomes may play an important role in *Entamobea*. DNA sequencing is considered the gold standard for identifying such mutations. Nevertheless, DNA sequencing method is cumbersome and costly when large numbers of samples need to be rapidly analyzed. The method, therefore, is not always suitable for use in epidemiological studies.

In the present study, PCR products of 16S-like rRNA gene of *E. histolytica*, *E. moshkovskii *and *E. dispar *were subjected to screening for mutation by riboprinting and SSCP analysis. These products were of a relatively small sizes, 553 bp for *E. moshkovskii*, 439 bp for *E. histolytica *and 174 bp for *E. dispar*, and were found to be particularly suitable for SSCP analysis.

Riboprinting analysis detected a mutation in only one of the *E. histolytica *samples, from a stool specimen (Stool- 5); the alteration in the RFLP pattern (i.e. the PCR product remained undigested) was due to substitution of G by A in the recognition site of *N1a*IV. The inability of riboprinting to detect mutations in a majority of *Entamoeba *isolates is due to the limitation that RFLP detects mutation only when they occur in restriction endonuclease cut sites. However, SSCP analysis detected mutation in a total of five *E. histolytica *and three *E. moshkovskii *isolates from stool and one *E. histolytica *isolate from saliva. This may be attributed to the ability of SSCP, unlike RFLP, to detect any mutation in a DNA sequence. The SSCP analysis results that were suggestive of mutations were reproducible (i.e. SSCP analysis showed identical profiles in three separate experiments).

In this study none of the 171 *E. dispar *isolates showed characteristics suggestive of mutation by SSCP analysis, perhaps due to the screening of only a 174 bp region of the gene. The chances of finding a mutation in such a small region is less than in larger regions studied for the other two species.

Although it was observed that riboprinting was useful for screening a large number of samples, especially when looking for a specific single nucleotide polymorphism, the method has some inherent disadvantages. In this method, a large amount of amplicon (15 μl) is needed for digestion with each of several enzymes, and each clinical sample ends up being electrophoresed on several gels, making it a very laborious and time consuming procedure.

SSCP analysis, on the other hand, was found to be simple and convenient in the present study. The results were obtained from only 2 μl of PCR product on a single SSCP gel, which in the long run turned out to be much more economical. The total time needed for PCR-SSCP was less than 24 hours. The SSCP technique developed and evaluated in our laboratory provides us with a rapid tool for detecting mutations in *Entamoeba*. This method could conveniently supplement other methods of mutation detection analysis on large-scale. The limitation of SSCP, although minor, is that it detects the occurrence of single base mutations in a segment of DNA but does not give any information on the type or location of the base changes, which have to be confirmed by nucleotide sequencing.

In the present study, the results showed that SSCP analysis was able to detect even single nucleotide differences (Figure 5, Figure 7, and Figure 8). Nucleotide sequences were verified three times by sequencing of products of different PCR reaction from the same source of DNA to rule out any discrepancy due to sequencing errors. The sequencing analysis identified four new *E. histolytica *genotypes and three new *E. moshkovskii *genotypes (Figure 4 and Figure 6). Recently, a report from Bangladesh [16] studied clinical specimens using six tRNA-linked STR loci, and detected 85 genotypes in 111 unrelated samples. Another report from Bangladesh [15] also studied clinical samples but used a nested PCR-RFLP of the SREHP gene. Twenty five genotypes among 42 intestinal isolates and 9 genotypes among 12 ALA samples were found. Out of 9 genotypes from liver abscess samples, 8 were unique to the ALA samples investigated. In our study no unique genotypes among any of the liver abscess samples and urine samples from ALA cases were found, but out of 5 *E. histolytica *genotypes from stool samples 4 were unique to the stool samples investigated. In addition, our study also detected one unique *E. histolytica *genotype from a saliva sample from an ALA case and three new *E. moshkovskii *genotypes from stool samples. Clark and Diamond [6] combined results of both PCR-RFLP of SREHP and amplification of the SSG locus to report 16 different genotypes among 18 isolates of *E. histolytica *from varied geographical locations. Haghighi et al. [11] reported a total of 53 different genotypes among 63 isolates of *E. histolytica*, mostly from Japan and Thailand, using sequencing of four loci (two tRNA-linked STR loci, chitinase, and SREHP). The high level of diversity reported from different geographic locations suggest that the rapid generation of new *Entamoeba *variants is taking place. The occurrence of new *E. histolytica *and *E. moshkovskii *genotypes in the area of Puducherry, India, may be a unique finding and the close relationship of these genotypes with a recognized human pathogen like *E. histolytica *should prompt further studies.

In this study, the presence of mutations was explored in a region of 16S-like rRNA gene of *E. histolytica*, *E. dispar *and *E. moshkovskii *by applying riboprinting and SSCP analysis. Further studies are needed to extend mutation detection to the complete ribosomal RNA gene of these species, which will possibly reveal much more about genetic variation in *Entamoeba*.

## Conclusion

The present study has revealed the subsistence of mutation in ribosomal RNA gene of *E. histolytica *and *E. moshkovskii*, which points towards the existence of intra-species genetic variation in *E. histolytica *and *E. moshkovskii *isolates infecting humans.

## Competing interests

The authors declare that they have no competing interests.

## Authors' contributions

SCP supervised and coordinated the study, and helped to draft the manuscript. KK carried out the experimental work, and drafted the manuscript.

## Pre-publication history

The pre-publication history for this paper can be accessed here:



## References

[B1] World Health Organization (1997). Amoebiasis. Wkly Epidemiol Rec.

[B2] Lösch FA (1975). Massive development of amebas in the large intestine. Translation from the original in Russian, 1875. Am J Trop Med Hyg.

[B3] Parija SC, Parija SC (2006). Amoebae: Intestinal Amoebae Pathogenic Free-living Amoebae. Text book of medical parasitology.

[B4] Clark CG, Diamond LS (1991). Ribosomal RNA genes of 'pathogenic' and 'nonpathogenic' *Entamoeba histolytica *are distinct. Mol Biochem Parasitol.

[B5] Bhattacharya S, Bhattacharya A, Diamond LS (1992). *Entamoeba histolytica *extrachromosomal circular ribosomal DNA: analysis of clonal variation in a hypervariable region. Exp Parasitol.

[B6] Clark CG, Diamond LS (1993). *Entamoeba histolytica*: a method for isolate identification. Exp Parasitol.

[B7] Sehgal D, Bhattacharya A, Bhattacharya S (1993). Analysis of a polymorphic locus present upstream of rDNA transcription units in the extrachromosomal circle of *Entamoeba histolytica*. Mol Biochem Parasitol.

[B8] Ghosh S, Frisardi M, Ramirez-Avila L (2000). Molecular epidemiology of *Entamoeba *spp.: evidence of a bottleneck (demographic sweep) and transcontinental spread of diploid parasites. J Clin Microbiol.

[B9] Zaki M, Clark CG (2001). Isolation and characterization of polymorphic DNA from *Entamoeba histolytica*. J Clin Microbiol.

[B10] Li E, Kunz-Jenkins C, Stanley SL (1992). Isolation and characterization of genomic clones encoding a serine-rich *Entamoeba histolytica *protein. Mol Biochem Parasitol.

[B11] Haghighi A, Kobayashi S, Takeuchi T, Thammapalerd N, Nozaki T (2003). Geographic diversity among genotypes of *Entamoeba histolytica *field isolates. J Clin Microbiol.

[B12] Haghighi A, Kobayashi S, Takeuchi T, Masuda G, Nozaki T (2002). Remarkable genetic polymorphism among *Entamoeba histolytica *isolates from a limited geographic area. J Clin Microbiol.

[B13] Simonishvili S, Tsanava S, Sanadze K, Chlikadze R, Miskalishvili A, Lomkatsi N, Imnadze P, Petri WA, Trapaidze N (2005). *Entamoeba histolytica*: the serine-rich gene polymorphism-based genetic variability of clinical isolates from Georgia. Exp Parasitol.

[B14] Rivera WL, Santos SR, Kanbara H (2006). Prevalence and genetic diversity of *Entamoeba histolytica *in an institution for the mentally retarded in the Philippines. Parasitol Res.

[B15] Ayeh-Kumi PF, Ali IM, Lockhart LA, Gilchrist CA, Petri WA, Haque R (2001). *Entamoeba histolytica*: genetic diversity of clinical isolates from Bangladesh as demonstrated by polymorphisms in the serine-rich gene. Exp Parasitol.

[B16] Ali IK, Mondal U, Roy S, Haque R, Petri WA, Clark CG (2007). Evidence for a link between parasite genotype and outcome of infection with *Entamoeba histolytica*. J Clin Microbiol.

[B17] Zaki M, Clark CG (2001). Isolation and characterization of polymorphic DNA from *Entamoeba histolytica*. J Clin Microbiol.

[B18] Pinheiro SM, Maciel RF, Morais MA, Aca IS, Carvalho LB, Coimbra MR (2005). Genetic characterization of *Entamoeba dispar *isolates in Northeast Brazil. Acta Trop.

[B19] Ramos F, García G, Valadez A, Morán P, González E, Gómez A, Melendro EI, Valenzuela O, Ximénez C (2005). *E. dispar *strain: analysis of polymorphism as a tool for study of geographic distribution. Mol Biochem Parasitol.

[B20] Sambrook J, Russell DW (2000). Molecular Cloning, A Laboratory Manual.

[B21] Khairnar K, Parija SC (2007). A novel nested multiplex polymerase chain reaction (PCR) assay for differential detection of *Entamoeba histolytica*, *E. moshkovskii *and *E. dispar *DNA in stool samples. BMC Microbiol.

[B22] Parija SC, Khairnar K (2007). Detection of excretory *Entamoeba histolytica *DNA in the urine, and detection of *E. histolytica *DNA and lectin antigen in the liver abscess pus for the diagnosis of amoebic liver abscess. BMC Microbiol.

[B23] Khairnar K, Parija SC Detection of *Entamoeba histolytica *DNA in the saliva of amoebic liver abscess patients who received prior metronidazole treatment. J Health Popul Nutr.

